# Multimodal Microscale Imaging of Textured Perovskite–Silicon Tandem Solar Cells

**DOI:** 10.1021/acsenergylett.1c00568

**Published:** 2021-05-28

**Authors:** Elizabeth M. Tennyson, Kyle Frohna, William K. Drake, Florent Sahli, Terry Chien-Jen Yang, Fan Fu, Jérémie Werner, Cullen Chosy, Alan R. Bowman, Tiarnan A. S. Doherty, Quentin Jeangros, Christophe Ballif, Samuel D. Stranks

**Affiliations:** † Cavendish Laboratory, University of Cambridge, 19 JJ Thomson Avenue, Cambridge CB3 0HE, U.K.; ‡ École Polytechnique Fédérale de Lausanne, Photovoltaics and Thin-Film Electronics Laboratory, Neuchatel 2002, CH, Switzerland; § Department of Chemical Engineering, Stanford University, Stanford, California 94305, United States; ∥ Department of Chemical Engineering & Biotechnology, University of Cambridge, Philippa Fawcett Drive, Cambridge CB3 0AS, U.K.

## Abstract

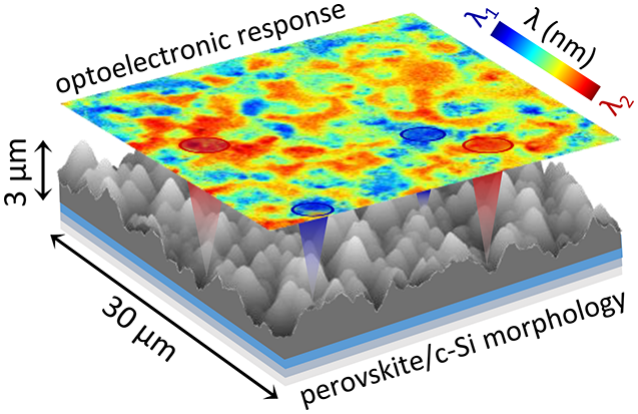

Halide perovskite/crystalline silicon (c-Si) tandem solar cells promise power conversion efficiencies beyond the limits of single-junction cells. However, the local light-matter interactions of the perovskite material embedded in this pyramidal multijunction configuration, and the effect on device performance, are not well understood. Here, we characterize the microscale optoelectronic properties of the perovskite semiconductor deposited on different c-Si texturing schemes. We find a strong spatial and spectral dependence of the photoluminescence (PL) on the geometrical surface constructs, which dominates the underlying grain-to-grain PL variation found in halide perovskite films. The PL response is dependent upon the texturing design, with larger pyramids inducing distinct PL spectra for valleys and pyramids, an effect which is mitigated with small pyramids. Further, optimized quasi-Fermi level splittings and PL quantum efficiencies occur when the c-Si large pyramids have had a secondary smoothing etch. Our results suggest that a holistic optimization of the texturing is required to maximize light in- and out-coupling of both absorber layers and there is a fine balance between the optimal geometrical configuration and optoelectronic performance that will guide future device designs.

Among the most promising candidates for the next generation of high-performance photovoltaic (PV) cells is a combination of a mature and reliable material (crystalline Si, c-Si) with one that is new and disruptive (halide perovskites) in the form of perovskite/c-Si multijunction solar cells. The most efficient single-junction c-Si devices are composed of micrometer-sized pyramids at both rear and front surfaces, which boost the absorption of photons in the c-Si absorber by reducing reflection losses and enhancing trapping of incident light in the high-index-of-refraction material.^1,2^ By depositing a halide-perovskite-based top cell directly on the pyramidal structure in a monolithic tandem device, c-Si preserves its high optical performance and the entire device stack more efficiently uses the sun’s light compared to single-junction solar cells.^3^ Halide perovskite semiconductors take an ABX_3_ crystal structure, where A is a monovalent cation (methylammonium, formamidinium (FA), and/or Cs), B is a divalent metal (typically Pb or Sn), and X is a halide (typically Br and/or I); the highest performance compositions have mixed components in their A and X sites.^4^ Although the nascent halide perovskite material class still faces a number of challenges, such as various performance heterogeneities^5−7^ arising from local defect distributions,^8−10^ and long-term stability issues,^11^ they have reached power conversion efficiencies (η) exceeding 25% in a single-junction solar cell with promising stability.^12,13^ When coupled into a tandem device, with c-Si as the bottom cell, the multijunction η exceeds 29%,^12,63^ surpassing the record single-junction c-Si solar cells (∼26.7%)^14^ and cementing this technology as extremely promising for commercialization.

One of the reasons why the staple c-Si solar cells are so efficient, in spite of their indirect bandgap, is the judicious design of the device architecture, including texturing, to achieve near-optimal light management. Therefore, the power output of any planar multijunction PV device with c-Si as the bottom cell will ultimately be limited (particularly the photocurrent) compared to the texturing design.^15−19^ Recently, the fabrication of halide perovskite top cells on the microscale c-Si pyramids using a variety of techniques has been reported, with the resulting perovskite absorber either deposited conformally^20−23^ or nonconformally.^24,25^ When the perovskite material is introduced onto the already complex multilayer stack, additional device optimization challenges arise, as the morphology of the light absorbing materials now consists of two multiscale, spatially varying components (i) the micrometer-sized c-Si pyramids and (ii) the <1 μm size polycrystalline grains of the perovskite. Each component adds discrete and localized heterogeneities to the optoelectronic properties. These heterogeneities can be probed through local photoluminescence (PL) measurements that assess radiative recombination events; any nonradiative recombination sites lead to voltage losses. In the operating cells, the external PL intensity from the cell at open circuit must be maximized to optimize power-conversion efficiency.^26^ Thus, there is a need to spatially resolve the PL to understand how the pyramids influence the radiative and nonradiative recombination processes, which ultimately dictate local voltage losses in solar cells.^27,28^ While many microscopy studies have been performed on c-Si^29−31^ and perovskite^32−37^ single junction devices, until recently, there have been few investigations that experimentally measure the local performance of the entire monolithic sample stack.^22,24^ By imaging the optoelectronic response of the perovskite within a high-performance textured perovskite/c-Si multijunction solar cell, we can investigate how texturing directly impacts the optoelectronic response and therefore, performance of the perovskite cell.

Here, we employ multimodal microscopy techniques in conjunction with correlative optical modeling to understand how the distinct micro- and nanoscale constructs affect local device performance in full perovskite/c-Si tandem solar cells. We capture the optoelectronic heterogeneities via wide-field, hyperspectral PL imaging, which allows us to resolve the radiative recombination events both spatially and spectrally in the conformal perovskite device layer on multiple c-Si texturing designs. Atomic force microscopy (AFM) maps acquired on the same scan area allow us to link the spatially heterogeneous PL response with the topographic variations of the sample. We reveal a distinct PL spectral heterogeneity pattern of the perovskite, which depends on the geometry of the c-Si texturing. In large (5 μm pyramids) texture schemes, the majority of the spatial intensity and spectral PL heterogeneities can be attributed to increased photon trapping within the pyramid valleys from the optical texturing, which provides a dominant contribution to the emission over any underlying intrinsic perovskite grain-to-grain PL heterogeneity. In designs with reduced pyramid sizes, the PL distribution is more homogenized. We show that performance parameters such as the PL quantum efficiency (PLQE) and the local quasi-Fermi level splitting (QFLS) values can be boosted by texture tuning. These results highlight the importance of the c-Si pyramid dimensions on the optoelectronic response of the perovskite and indicate that the perovskite emission and performance is tunable depending on the texturing design. Our work presents the need to construct light-absorbing materials in which the photon in- and out-coupling is holistically optimized to increase multijunction device performance.

We fabricate perovskite/c-Si textured tandem solar cells in the architecture shown in Figure 1a using methods reported in our previous work^20^ (see methods section). The denoted 5 μm pyramidal texturing is formed by etching a monocrystalline n-type (100) Si wafer with a potassium hydroxide base, which will preferentially etch along the (111) planes.^38^ The perovskite employed here is (Cs_0.18_FA_0.82_)Pb(I_0.87_Br_0.13_)_3_, deposited using a two-step process: (i) PbI_2_ is coevaporated with CsBr, followed by (ii) dynamic spin-coating of a FABr/FAI mixture to convert to a perovskite layer with a ∼1.6 eV bandgap, ascertained from external quantum efficiency and UV–vis measurements in Figures S1 and S2, respectively (Supporting Information). Figure 1b displays a top-view scanning electron microscopy (SEM) image of the 5 μm, c-Si texturing sample morphology, where randomly distributed pyramids decorate the front surface of the device. The inset SEM image highlights the conformal coating of all the layers on top of the textured c-Si (we assume the perovskite layer is of uniform thickness across the sample, as the same fabrication process is employed here as in ref (20)). The height distribution of the pyramids in this design measured with AFM is shown in Figure 1c (see methods), with a peak height of up to 5 μm. Representative macroscopic light current density–voltage (*J–V*) curves are shown in Figure 1d, confirming the electrical performance in both reverse and forward bias of the full solar cell (η > 23%).

**Figure 1:**
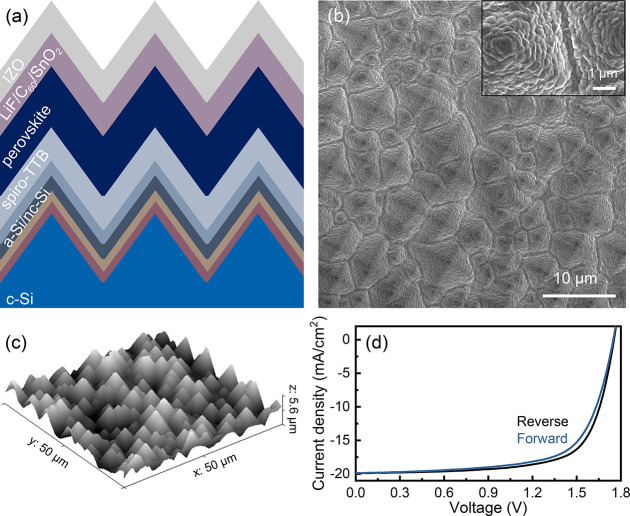
(a) Cross-sectional schematic of the different layers within the two-junction tandem device (not to scale). The a-Si/nc-Si layers consist of a-Si:H(i)/a-Si:H(n)/nc-Si:H(n^+^)/nc-Si:H(p^+^) from bottom to top. (b) Top-view scanning electron microscopy (SEM) secondary electron (SE) image of a perovskite/c-Si tandem cell, showing the pyramid texturing and size distribution. The inset SE-image highlights the conformal perovskite coating. (c) 3D-view of a 50 × 50 μm^2^ atomic-force microscopy (AFM) map of Si pyramids; the pyramids have a peak height of up to ∼5 μm and an average base diameter of ∼7 μm. (d) Forward and reverse light *J–V* curves of a typical perovskite/c-Si tandem textured solar cell used in this work. Figures of merit, forward: *V*_oc_ = 1.75 V, *J*_sc_ = 19.9 mA/cm^2^, FF = 68.2%, η = 23.70%. Figures of merit, reverse: *V*_oc_ = 1.75 V, *J*_sc_ = 19.9 mA/cm^2^, FF = 70.9%, η = 24.7%.

To investigate how this 5 μm texturing influences the optoelectronic properties of the complex device stack, we image the local photoluminescence (PL) response of the perovskite with a wide-field hyperspectral optical microscope (Figure 2; for setup details see methods section and Figure S3, Supporting Information). In Figure 2a,b, we show the PL intensity maps corresponding to two emitted wavelengths (λ_em_), 765 and 785 nm (1.62 and 1.58 eV), respectively, of the perovskite absorber with a bandgap of 1.59 eV (Figure S2b, Supporting Information). From these images, we observe unique PL spatial distributions for each photon emission energy (see Figure S4 for an RGB image with Figure 2a,b overlaid, Supporting Information). The spatial λ_em_ dependence of the perovskite on textured c-Si is demonstrated by plotting two local PL spectra (corresponding to the regions with the rose and gray-blue circles in Figure 2a–d), in Figure 2e, along with the average PL emission spectrum of the entire region (black). This is further confirmed by the λ_em_ peak maximum map (Figure 2d), which shows a large spatial variation in the PL peak. Together, these results highlight the spatial and spectral heterogeneity of the PL peak maximum of the perovskite surface on the textured tandem solar cell. This variation is beyond the spatial scale that would be expected for grain-to-grain PL distributions (submicron) in the neat perovskite films. To confirm this, we show a hyperspectral λ_em_ peak maximum map of the same perovskite material on a planar c-Si substrate (Figure 2g and Figures S5 and S6, Supporting Information), which has features dominated by grain-to-grain variations due to spatially dependent trap distributions.^8,39^ Moreover, the planar perovskite has fewer local variations over all of the PL peak maximum. We note that the underlying perovskite emission variations are visible yet largely insignificant in the textured device stack (Figure 2d). These results suggest that there is a strong influence of the textured substrate on the perovskite emission properties, which dominates the PL heterogeneity.

**Figure 2:**
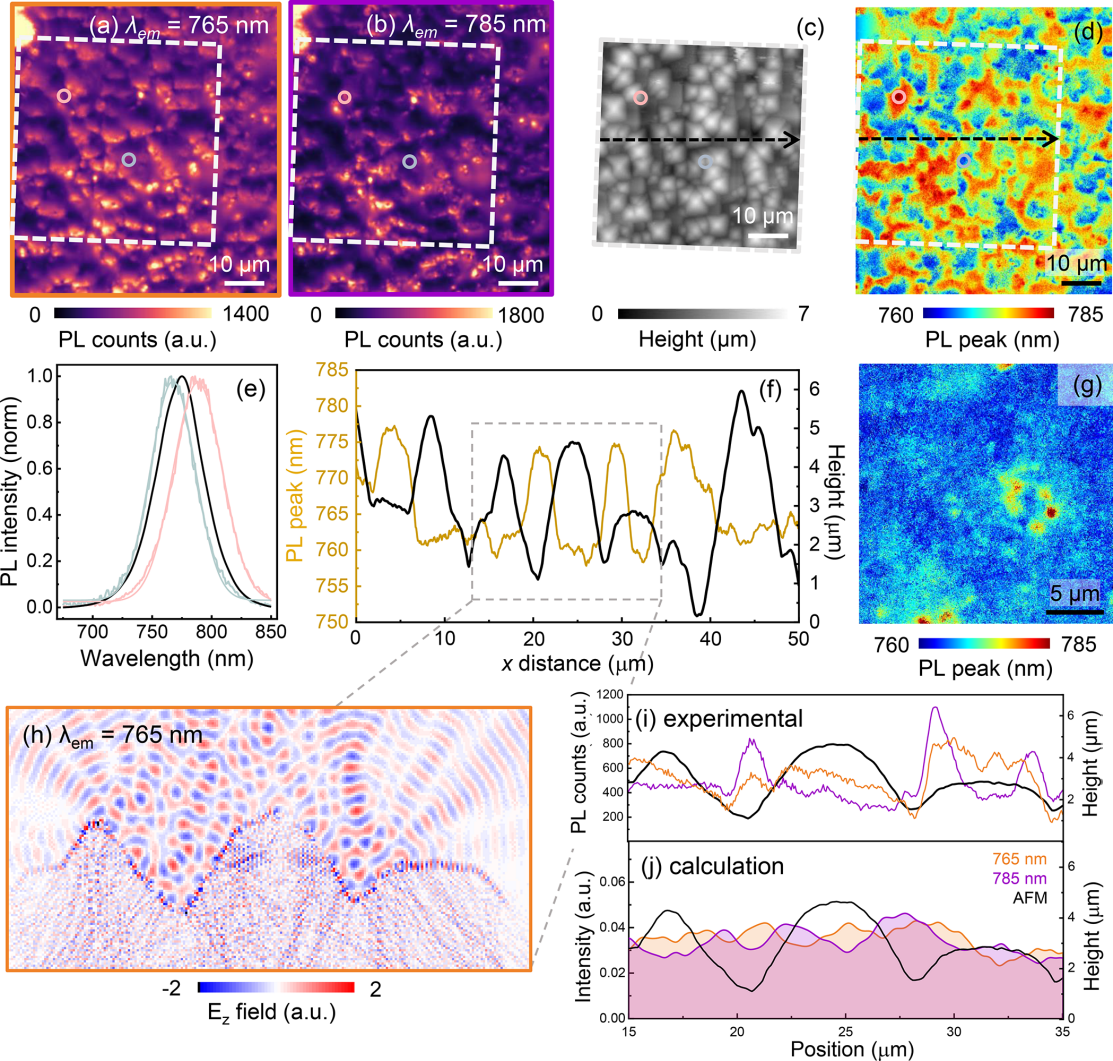
Wide-field hyperspectral PL maps of the perovskite emission on a 5 μm c-Si texturing scheme at (a) λ_em_ = 765 nm and (b) λ_em_ = 785 nm on the same region. The dashed white box indicates the region of the AFM map. (c) 50 × 50 μm^2^ AFM image on the same denoted region. (d) λ_em_ peak maximum map, where blue areas have a shorter λ_em_ (higher energy) peak maximum and red regions signify a longer λ_em_ (lower energy). This map was extracted by performing a smoothing Gaussian filter on the λ-axis of the hyperspectral image, using the full-width half-maximum (fwhm) of each local PL spectra, prior to extracting the λ_em_ maximum (see methods). (e) Normalized spatially averaged (black) and local (gray-blue and rose) PL spectra from the maps in panels a and b, where the local spectra correspond to the circles in panels a–d. (f) Height profile of the AFM (black) and the PL peak maximum (gold curve, smoothed by applying a 12-point curve average), from the same sample location. The line traces are taken along the black line, in panels c and d (*x* travels left to right, arrow direction). (g) λ_em_ maximum map of a planar perovskite film on a flat c-Si substrate. The PL maps were acquired in ambient conditions with a 100× objective, 405 nm incident light (λ_exc_), and illumination flux of (a, b, and d) 2150 mW/cm^2^ and (g) 300 mW/cm^2^ (see Figure S11, Supporting Information, for the map taken with an excitation intensity comparable to that in panel g, no discernible difference in the spatial distribution with flux). (h) Correlated FDTD simulation of perovskite photon emission that shows the results for calculations with point emitters as λ_em_ = 765 nm. See Figure S12 for results with point emitters as λ_em_ = 785 nm, Supporting Information. Comparison of light emission detected via (i) optical microscopy (PL) and (j) FDTD calculation for intensity, *I* (see eq 1 and Experimental Methods), of the perovskite emission λ_em_ = 765 (orange) and 785 nm (purple), with the black lines representing the measured morphology from AFM. The calculated curves in (j) are the result of 19 emitters detected in the far field (Figures S12a and S13a, Supporting Information), distributed near the perovskite surface.

To understand how the spatially heterogeneous PL output is related to the texturing design of the pyramids, we perform AFM imaging on the same scan area as the wide-field PL measurements (Figure 2c). The white-dashed box in Figure 2a,b,d denotes the 50 × 50 μm^2^ region where the AFM map was acquired. These multimodal correlations between microscope rigs were made by scribing a fiduciary mark on the sample (the edge of the mark is seen in the top-left corner of the PL images). In Figure 2f, we plot line traces of the AFM height (black) against the PL peak maximum position (gold) (see Figure S7 for additional slices, Supporting Information), revealing that the shorter wavelength PL emission closely follows the topography height, while the lower energy PL is more prevalent in the valleys and sidewalls of the textured landscape. We note that the spatial λ_em_ dependence is not an artifact of the optical focus, even in spite of the large height variations (see Figures S8 and S9 for measurements at different focal planes, Supporting Information) or objective collection efficiency (see Figure S10 for results with different objective lenses, Supporting Information). These results strongly indicate that the observed PL spectral and spatial heterogeneity primarily arise from the texture variations; this conclusion is further emphasized below when we consider different texturing schemes.

By implementing finite-difference time-domain (FDTD)^40^ simulations with the open-source software package MEEP (see methods for computational parameters), we discern how this texture type may lead to such specific and local PL heterogeneity phenomena.^41^ Selecting FDTD simulations for the optical modeling enables us to account for the thin film, textured, and localized nature of the perovskite layer. Ray tracing is incompatible with layers thinner than the wavelength of interest, and transfer matrix methods are limited primarily to planar film applications. Although there have been reports using a combination of these two methods,^42^ the results focus on macroscopic absorption/reflection spectra and do not provide insight on the local texture information. FDTD simulations cope with both textured and thin layers while also providing a cross section of the local electric field intensity within a sample of interest albeit often with long computational times.

In Figure 2h, we use the AFM line trace from Figure 2f as an input for the MEEP code to model the morphological variation, with a 2D FDTD model employed for the 2D line scan. In this simulation, we emulate the wide-field PL experimental conditions and detect the emission arising from radiative recombination of charge carriers within the perovskite layer by running two λ_em_ simulations where we position numerous emission point sources along the perovskite film surface (denoted in the Figure S12a, Supporting Information): one simulation for all emission points λ_em_ = 765 nm (Figure 2h), the other with all emission points λ_em_ = 785 nm (Figure S12, Supporting Information). We subsequently monitor the emission intensity in the far field (red dashed line in Figure S12b, Supporting Information) once the calculation has reached a steady state (see methods). By placing an FDTD monitor tens of microns away from the perovskite surface (Figure S12b, Supporting Information) we capture the superposition of a set of scattered waves at a finite number of diffraction orders, equivalent to the far-field response. The MEEP modeling simulates the magnitude of the *E*_*z*_ field, which is related to the intensity *I* by
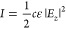
1where *c* is the speed of light and ε is the permittivity. Figure 2i shows the experimentally obtained PL emission intensity along the sample cross-section, while Figure 2j compares the intensity given by the FDTD calculations, for the λ_em_ = 765 nm (orange) and 785 nm (purple) curves. In Figure 2i,j we correlate the emission intensity of the two wavelengths and reveal a qualitative agreement between the experimental PL results and the FDTD simulations. The 765 nm emission is the dominant signal near the pyramid apex whereas the 785 nm emission is enhanced along the sides and within the valley of the pyramids, consistent with our experimental measurements. We prevent discrete, constructive fluctuations in the calculation curves and emission angular distributions by simulating one emitting source at a time and then adding the intensities from all 19 asynchronous emitters together to reproduce the PL collected by the objective lens which is diffuse and incoherent (Figure S13 includes the results comparing the synchronized vs asynchronous point emitters along with the experimental data, Supporting Information). The local fluctuations are not an artifact of noise and are distinctly caused by the texture, which we demonstrate by monitoring the perovskite PL out-coupling on planar perovskite/c-Si surfaces (Figures S14 and S15, Supporting Information). The photon intensity response of the planar surfaces are much more spatially uniform, confirming that the detected photon emission is highly dependent on the texturing structure.

To describe PL enhancement at the side walls, light reflected in this region contributes to the intensity at a detector in the far-field and leads to an enhancement in apparent emission from the valleys with respect to other regions. Such enhancements will depend sensitively on the angle of the side wall and the spatial origin of the emitted light along the wall (Figure S16, Supporting Information). However, a fraction of the light hitting the side wall will be transmitted into the top absorber (perovskite) material. Light corresponding to λ_em_ = 765 nm will be readily reabsorbed by the perovskite owing to the ∼1.6 eV bandgap (onset at 775 nm) and strong absorption coefficient^43^ (Figure S2 for absorption spectrum and Table S1, Supporting Information). Such excitations will likely not be re-emitted^44,45^ as most will be lost through nonradiative recombination given the voltage of the top cell device (∼1.05 V) indicates that external luminescence yields are ≪1%. By contrast, light corresponding to 785 nm is below the bandgap of the material and will not be significantly absorbed (Figures S1 and S17, Supporting Information); this light can thus re-emerge from the film following reflection and transmission events and contribute to the intensity measured by the detector at that region. The net effect is an enhancement of λ_em_ = 785 nm intensity compared to 765 nm in the regions corresponding to the valleys and side walls, as observed in the experimental measurements and simulations. Near the peaks, there is less opportunity for additional interaction of the emitted light with the material, and thus there is less dependence on the wavelength from those regions. Moreover, optical field enhancement hot-spots at the c-Si peaks have been previously shown, and these may influence the emission of the overlaid perovskite in these regions.^46^ Other optical phenomenon, such as a variable local density of photon states, could also contribute to the observed spatial dependence of the PL.^47^

We note that the observed effects may also be exaggerated by any local chemical (halide) segregation in the perovskite leading to bandgap heterogeneities. Indeed, local chemical mapping on 5 μm textured sample reveals variations in the halide distribution, suggesting there is slightly more Br in the pyramid valleys (Figure S18). However, if chemical variations alone explain the observed PL differences, we would in fact expect a blue-shifted emission in these Br-rich valleys with respect to the remaining material, in contrast to what is observed (cf. Figure 2f). Thus, this provides further evidence that the texturing dominates the optoelectronic response of the perovskite. We note that typical chemical variations in the planar mixed halide perovskite films are on smaller length scales (i.e., hundreds of nanometers, cf. Figure 2g), and thus the small variations in halide distribution induced by the texture will also need to be considered when optimizing composition and thickness for overall device optimization. Any vertical chemical segregation in the perovskite will further exaggerate the effects; for example, a thin iodide-rich surface layer would lead to a lower emission energy from the surface, but this emitted light would not be readily reabsorbed because the bulk of the material is at a higher bandgap.^48^ Note, in our previous work,^20^ minimal chemical variation was previously measured throughout the conformal perovskite layer on the textured-c-Si. We also note that the slightly enhanced absorption of the excitation in the valleys and sidewalls will contribute to the observed emission heterogeneity but cannot explain the effects alone.

To investigate how the optoelectronic behavior of the perovskite layers can be tuned through the underlying c-Si texturing, we consider three different texturing schemes in Figure 3. Specifically, we consider the perovskite deposited on three different c-Si etches: (i) the 5 μm pyramid texturing scheme (violet, Figure 3a,d,g) using the same KOH etching process as the tandem sample analyzed thus far; (ii) 5 μm + smooth texture (red, Figure 3b,e,h), where a second etching treatment is performed on the 5 μm pyramids; (iii) a ∼2-μm-sized pyramid etch produced by KOH etching (green, Figure 3c,f,i). AFM line traces for these samples are shown in Figure S19, Supporting Information. By comparing the respective PL peak maximum maps, Figure 3d–f, we immediately identify that the PL spectral variation across the samples generally decreases when we smooth the pyramids and even more dramatically when we decrease the average pyramid size. These results are emphasized further when considering the line scans from left to right in Figure 3g–i, confirming the earlier results that the texturing from the large pyramids dominates the PL response of the overlaying perovskite cell. Again, the texture causes a nonrandom PL peak shifting pattern (identified in Figure 2), while the small pyramids trend toward a more homogenized emission (closer to that of the planar sample, see Figure S6, Supporting Information).

**Figure 3:**
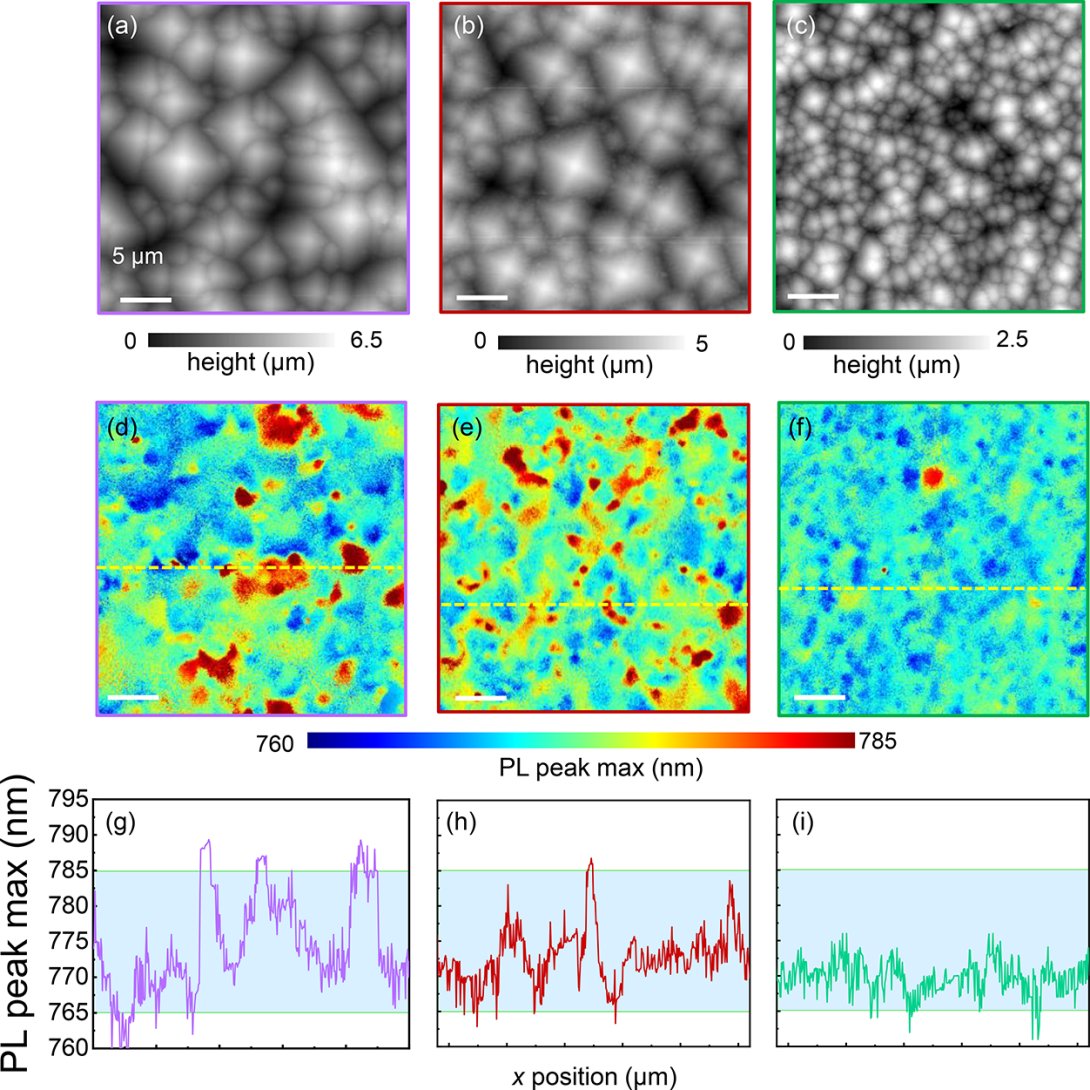
(a)–(c) 30 × 30 μm^2^ AFM maps of the three different perovskite/c-Si texturing schemes displaying the morphology of the 5 μm, 5 μm + smooth, and 2 μm geometries, respectively. (d)–(f) 30 × 30 μm^2^ PL peak maximum maps extracted from wide-field hyperspectral PL maps, λ_exc_ = 405 nm illumination at 5 sun photon flux intensity and a 100× objective (see Figure S5, Supporting Information). Here, the PL peak variation is a measure of heterogeneity (g)–(i) PL peak maximum line traces extracted from slices denoted by the yellow lines in panels d–f. Long tick lines in panels g–i correspond to 5 μm steps.

We quantitatively summarize the performance differences between the multiple texturing geometries in Figure 4 by measuring the absolute photon counts from the perovskite in each sample. The quasi-Fermi level splitting (QFLS), which is directly related to the open-circuit voltage of a solar cell,^29^ is extracted by fitting the local absolute PL peaks. By fitting the entire peak at each spatial point (see methods),^49^ we extract and subsequently spatially map the local QFLS of the perovskite absorber of full devices (Figure 4a–c). The spatial variation of the QFLS reflects the spectral (Figure 3d–f) and intensity (Figure S5, Supporting Information) variations including the dominant influence of the texture. We find that the 5 μm + smooth has the largest QFLS values, where the peak of the histogram (fit by a Gaussian distribution curve in Figure 4d) is 1.190 eV with a narrow distribution (fwhm of 0.024 eV). The 5 μm sample QFLS distribution has a peak at 1.176 eV but has a 2× wider distribution of 0.042 eV, consistent with the larger spatial heterogeneity in PL properties. The sample with smaller (2 μm) pyramids has the lowest QFLS peak of 1.172 eV and fwhm of 0.029 eV. See the additional trace including the planar QFLS measurements in Figure S20, Supporting Information. These local distribution trends match closely to macroscopic PLQE measurements on these three samples (Figure 4e), with the largest PLQE value seen for the 5 μm + smooth geometry. The direct visualization of the QFLS differences and agreement between the micro- and macroscopic measurements strengthens our conclusions that the photovoltaic performance can be tuned by changing the underlying c-Si structure, and the luminescence mapping methods provide ready feedback on these relationships. Voltage losses of even tens of millivolts between regions observed here will become very important as the perovskite layers of the tandem cells approach their radiative limits, as is the case with III–V absorbers. As such, these techniques will guide further strategies to control radiative and nonradiative recombination, for example, through passivation^50^ or photodoping effects.^51^ While additional passivation layers or additives may improve performance, excess nonperovskite material could lead to phase and/or operational instabilities.^52^ Therefore, one will need the careful and precise feedback on the true fractions of nonradiative recombination provided by the measurements presented here to guide these activities. We note that full device validation with the different textures would require further reoptimization of the perovskite process parameters and of the transport and recombination layer thicknesses for each texturing scheme and is beyond the scope of this current work.

**Figure 4:**
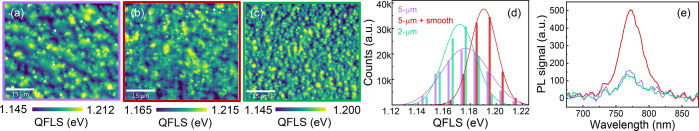
(a)–(c) Quasi-Fermi level splitting (QFLS) maps of the three perovskite/c-Si texturing schemes quantitatively displaying the local performance difference of the (a) 5 μm, (b) 5 μm + smooth, and (c) 2 μm geometries. The values were extracted from fits to local absolute PL spectra. (d) Histogram of the QFLS values for each of the three etching schemes based on the maps in (a)–(c). The 5 μm + smooth sample has the highest and narrowest QFLS distribution, while the 5 μm sample has the widest distribution of QFLS, signifying the greatest spatial heterogeneity. Each histogram was individually fit with a Gaussian peak with an *R*^2^ value of >0.99 in all cases. (e) Macroscopic relative PL quantum efficiency (PLQE) response of the three samples indicating that the performance trend is in agreement with the QFLS; see methods.

The influence of the texturing scheme on light in-coupling, out-coupling, and trapping in different types of solar cell materials has been calculated and discussed extensively in the literature.^18,19,53−57^ The method with which light interacts with a surface and its geometry will impact how and where it is subsequently absorbed/re-emitted. Total internal reflection and light trapping are two parameters that can be manipulated on the basis of the surface geometrical configuration, which we find is exaggerated with larger pyramid sizes. This is because the interaction of emitted light with the textured materials, through either subsequent absorption, reflection or both, will occur more prominently in the valleys than at the peaks, which will not exhibit such enhancement effects, exacerbated via larger pyramids. We acquire reflection measurements at normal incidence where *R* = 4% in the PL emission range, Figure S21, Supporting Information. The reflection response variation between the different texturing schemes is also negligible and the reflectance remains low (Figure S22, Supporting Information).

The difference between the length scales of perovskite PL intensity variations in the planar film (grain-to-grain, hundreds of nanometers) compared to the textured devices (∼2–10 μm) under the same illumination intensity is stark (cf. Figures 2, S5 and S6, Supporting Information). These results highlight the influence of the texture on the perovskite’s external emission profiles both in spectral properties and magnitude–consistent with good agreement between experimental and theoretically calculated PL variations shown in Figure 2. Therefore, there is an intricate relationship between the dimensions of the texturing within the solar cell stack, and the overall optical properties of both the top and bottom light-absorbing materials. Recognizing that the external luminescence of the perovskite is dominated by the geometrical configuration of the underlying c-Si bottom cell provides a tuning knob to control the photon out-coupling through modifying the texture scheme, for example the c-Si pyramid size, as verified in Figure 3.

Furthermore, the spectral emission variations depend on the perovskite bandgap (and any local variations thereof) as this parameter governs photon reabsorption. Thus, rational design of the bandgap or local bandgap variations^48,51^ could either reduce or emphasize the spectral heterogeneity depending on the desired application; for example, controlling the out-coupling wavelength in light-emitting diodes or trapping photons for enhanced light harvesting. The perovskite thickness will also play a crucial role in photon absorption and subsequent PL emission. Another important aspect when designing the texture parameters for optimal η is the homogeneity of light absorption. Due to spatially dependent light trapping in such texture schemes, it may be that certain regions of the solar cell receive lower concentrations of light and may act as local shunt resistors for the parts receiving more light, thus decreasing efficiency; alternatively, the regions with lower intensity may suffer from local regions where charge traps are not sufficiently saturated. The emission “hot spots” in the larger pyramid designs indicate higher concentrations of carriers in certain regions, which may enable local saturation of traps and thus texture can assist in maximizing the fraction of radiative recombination in regions where most recombination occurs; such effects may be beneficial as maximum PLQE in typical halide perovskites occurs at effective carrier densities equivalent to up to ∼10 suns.^9,51^ Each design characteristics discussed here relates back to the device performance, with an ultimate optimization requirement for maximizing light harvesting and external luminescence. One must therefore focus on holistic designs of the device by considering both the silicon and perovskite layers, not each in isolation. The optimization of the PV architecture along with the characterization techniques implemented here will be a useful platform for understanding effects that may occur in operation such as further defection formation and delamination in the conformal perovskite layer, and how these changes influence the overall device performance.

To summarize, we spatially and spectrally resolve the optoelectronic response of perovskite layers conformally coated and embedded within textured perovskite/c-Si multijunction solar cells. We experimentally show that low-energy emission (785 nm) is enhanced within the pyramid valleys and side walls, while the high-energy (765 nm) emission consistently tracks the surface morphology. By correlating the local topography with both PL measurements and FDTD simulations, we reveal that such a spatial and spectral dependence is primarily due to photon manipulation caused by the large geometrical constructs, which overshadows the underlying grain-to-grain PL variation in the perovskite absorbers, evidenced by measuring the PL in multiple c-Si texturing geometries. We find that in larger pyramids the perovskite PL spectral intensity and Quasi-Fermi-level splitting response are dominated by the underlying texture, while smaller pyramids are less susceptible to these effects. This phenomenon will play a role in the optimization of perovskite/c-Si tandem devices, and these results highlight the requirement to apply a comprehensive design plan to control the light in- and out-coupling of both light-absorbing layers in the tandem stack, as well as the opportunity to tailor structures for the further optimization of solar cells and other optoelectronic devices.

## Experimental Methods

### Solar Cell Fabrication

Silicon heterojunction (SHJ) bottom cells were fabricated using n-type double side-textured silicon float-zone wafers with a resistivity of 1–5 Ω and a thickness of 260 μm before texturing. For double-side textured (DST) SHJ, the wafers were immersed in a KOH-based solution, for 20 min (small pyramids, 2 μm) and 60 min (big pyramids, 5 μm), to obtain different pyramid sizes. Smoothening of the pyramids was done separately by immersion in a solution of nitric acid (HNO_3_), acetic acid (C_2_H_4_O_2_), and hydrofluoridric acid (HF) for 30 s (5 μm + smoothed pyramids). After that, the wafers were washed with deionized water. Intrinsic and doped hydrogenated amorphous silicon layers were deposited on both sides (n-type contact at the front and p-type contact at the back, respectively) of the wafer in a plasma-enhanced chemical vapor deposition (PECVD) reactor to passivate the silicon surface and to create the carrier-selective contacts. The back contact is made of a sputtered indium tin oxide (ITO)/Ag stack. The front side was capped by the recombination junction, which consisted of an n-type/p-type nanocrystalline silicon stack deposited in the same PECVD reactor as the amorphous silicon layers. For symmetrical samples (silicon wafer used as an electrode), n-type doped hydrogenated amorphous silicon layers are used on the rear side of the silicon wafer sides (instead of p-doped layers for SHJ samples).

Perovskite solar cells were fabricated in the p-i-n configuration. First, 12 nm of spiro-TTB was thermally evaporated as the hole transport layer. The perovskite absorber was then deposited using a hybrid sequential two-step method: first, PbI_2_ was coevaporated with CsBr onto the SHJ bottom cell. Then a mixture of formamidinium bromide and iodide (FAI:FABr 3:1, 0.513 M in ethanol, Greatcell Solar Materials) was dynamically spin-coated onto this template layer (4000 rpm during 30 s) in an N_2_ filled glovebox. The PbI_2_/CsBr template was 180 nm for flat substrates and 240 nm for textured tandems. The layers were then annealed at 150 °C for 30 min in ambient air (RH 30–40%) to crystallize the perovskite absorber. A layer stack of LiF (1 nm)/C60 (15 nm) was then thermally evaporated as the electron-selective layer. Afterward, a buffer layer of SnO_2_ (10 nm) was added via atomic layer deposition and indium zinc oxide (IZO) via sputtering (using a 90% In_2_O_3_ + 10% ZnO target, 100 nm); 120 nm of Ag was evaporated to form the front metal grid. All the layer thicknesses mentioned above were measured with respect to substrate plane (either a pyramid edge in the case of a textured cell or the horizontal for flat reference).

### Macroscopic *J–V* Curves

In-house *J–V* measurements were obtained on a temperature-controlled vacuum chuck at 25 °C, using a two-lamp (halogen and xenon) class AAA WACOM sun simulator with an AM1.5G irradiance spectrum at 1000 W/m^2^. Shadow masks were used to define the illuminated area (1.42 cm^2^). The cells were measured with a scan rate of 100 mV/s in air.

### Scanning Electron Microscopy

The SEM image displayed in Figure 1 was acquired with a Nova NanoSEM in SE mode at a magnification = 2000×. The working distance = 5.6 mm and the accelerating voltage = 5 kV.

### Atomic Force Microscopy

The AFM topography maps (512 × 512 pixels) were acquired on a Bruker Icon microscope in noncontact mode with a PtIr-coated Si AFM probe (resonance frequency = 75 kHz; spring constant = 3.0 N/m; tip radius = 25 nm).

### Hyperspectral Photoluminescence Microscopy

In this experiment, a 405 nm laser is normally incident on the sample, with a spot size of approximately 150 μm in diameter. The spatial resolution of the Photon Etc IMA microscope is diffraction limited (i.e., ≈500 nm in this case). A spectral volume Bragg grating is placed before the camera in order to detect only specified wavelengths, with a spectral resolution of 2.5 nm. The sample stage is immobile during data acquisition while the collection wavelength (λ_em_) is swept during the measurement. The incident photon flux used in Figure 2 was 2150 and 300 mW/cm^2^ for the textured and planar samples, respectively. Another hyperspectral map, with a power density of 100 mW/cm^2^ is shown in Figures S6 and S10 for the textured tandem solar cell (Supporting Information), where the PL spatial distribution is equivalent to the map with a higher photon flux, but with a lower signal-to-noise ratio. The PL peak maximum map in Figure 2d was smoothed to increase the signal-to-noise ratio, using a spectral Gaussian noise filter (=5, which equals the number of nearest neighbors’ points used to smooth the curve). For Figure 3 the incident photon flux was 300 mW/cm^2^. We measured the PL in a wide range of wavelength ranges and detected no other peaks, confirming there is no contribution to the PL from the nc-Si layer.

### Chemical Imaging

Nanobeam X-ray fluorescence (nXRF) data were acquired at the synchrotron beamline I14 of the Diamond Light Source. For all experiments, a 20 keV monochromatic X-ray beam (λ = 0.619 nm) was used, which was focused to under 100 nm. nXRF was measured with a four-element Si drift detector. Energy dispersive X-ray spectroscopy (SEM-EDX) data was acquired on an FEI nova nano SEM operating at 30 kV. Both nXRF and SEM-EDX data were analyzed in the python package hyperspy for multidimensional data analysis.

### Quasi-Fermi Level Splitting Fitting

The quasi-Fermi level splitting (QFLS) can be extracted from absolute calibrated PL spectra using the generalized Planck law.^58,59^ We fit the entire PL peak using the theory described by Katahara and Hillhouse.^60^ Briefly, the PL spectra is a function of energy *I*_PL_(*E*) written as
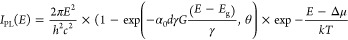
where *h* is Planck’s constant, *c* is the speed of light, α_0_*d* is the product of the film thickness *d* with a material dependent parameter that depends on the oscillator strength of the material, γ is the energy broadening factor of the below bandgap absorption tail, *E*_g_ is the bandgap energy, θ is the power of the below bandgap exponential tail (when θ = 1, γ is the Urbach energy), is a convolution integral between the below bandgap absorption tail and the above bandgap density of states for which a lookup table has been provided by Katahara and Hillhouse, *Δμ* is the QFLS, and *k* is the Boltzmann constant. The temperature of the sample is fixed to 300 K and α_0_*d* is fixed to 10, as suggested by Braly et al.^61^ Trial values for the remaining parameters: γ, *E*_g_, θ, and *Δμ* are guessed for average spectra of an entire area. The parameters are fit without bound using a Levenberg–Marquardt nonlinear least-squares fitting algorithm in Python. The optimized fits are inspected manually for quality and accuracy; then these input parameters are used for the inputs for the automatic fitting procedure for the entire map.

### PLQE Measurements

Photoluminescence measurements of the stacks were recorded using an integrating sphere, following the three-measurement approach of De Mello et al.^62^ Samples were illuminated by continuous wave temperature controlled Thorlabs 405 nm laser and excitation fluence varied with an optical filter wheel. The emission was recorded using an Andor IDus DU420A Silicon detector. Spot size was recorded using a Thorlabs beam profiler, where the size was set to be to where the intensity of the beam falls to 1/e^2^. All samples were illuminated in the same way and absorbed the same fraction of incident light. It is noted that these reported PLQE values are limited by reabsorption of emitted photoluminescence in other regions of the device.

### Finite-Different Time-Domain (FDTD) Simulations

The optical parameters for this perovskite are from ref (43), and a table of their values is listed as Table S1 (Supporting Information). In the simulation, only the perovskite and Si materials were included, as the hole and electron transport materials are much thinner than the photon wavelength of interest and do not strongly absorb in this part of the electromagnetic spectrum and are therefore negligible. We experimentally demonstrate this in Figure S23Supporting Information, where we perform hyperspectral PL measurements of a bare perovskite conformally coated 5 μm texture vs a full perovskite with the electron transport layers and IZO on top. The spectral heterogeneity distribution is nearly equivalent in both cases. The perovskite layer thickness = 430 nm and the spatial resolution in the simulation space was dependent upon λ where grid size = 24/(λ (in μm)). The cell area was 50 × 50 μm^2^ and its boundaries consisted of perfectly matched layers in order to avoid artificial reflections and the *E*_*z*_ field intensity was monitored. We also calculated the *E*_*x*_ and *E*_*y*_ directions and found their contributions negligible. Two types of simulations were performed, (i) Excitation: here, λ_exc_ = 405 nm is incident on the perovskite surface and the observed *E*_*z*_ field relates to how the incident light is absorbed within the multijunction layer stack. (ii) Emission: for this, 19 point emitters of λ_em_ = either 765 or 785 nm (enough to cover three pyramid peaks and two valleys) were positioned near the top surface of the perovskite layer. Nineteen different calculations were performed (one for each emitter), and the results were subsequently added together to obtain the final plot in Figure 2j. Upon reaching steady state, a line detector is placed >20 μm away from the perovskite surface to collect the far field light from the point emitters. The convergence of FDTD simulations occurs when the time-step (=50).

## Supporting Information Available

External quantum efficiency measurement of perovskite/c-Si tandem device; UV−vis spectrum and Tauc plot of perovskite; hyperspectral microscope schematic; photoluminescence (PL) microscopy overlays; comparison of planar and textured PL intensities; additional PL line scans; focal plane dependency measurements; objective lens dependency measurements; low illumination flux PL map; Finite different time domain (FDTD) calculations of λ_exc_ = 405 nm and zoom in FDTD for λ_em_ = 785 nm; synchronous vs asynchronous simulation for λ_em_ = 765 and 785 nm; planar vs textured FDTD simulations; FDTD of point emission profiles; chemical microscopy maps via energy-dispersion x-ray and nanobeam X-ray fluorescence; atomic force microscopy line traces; quasi-Fermi level splitting histograms; reflectance spectra; reflectance of tandems; table of optical parameters; PL peak maximum maps full devices vs perovskite film. (PDF)
