# Supplementary material for: Multimodal Microscale Imaging of Textured Perovskite–Silicon Tandem Solar Cells
Source: ACS Energy Lett. 2021 May 28;6(6):2293–304. doi: 10.1021/acsenergylett.1c00568 (PMC8291767; doi:10.1021/acsenergylett.1c00568)
Supplement: Supplementary file 1 — nz1c00568_si_001.pdf [file nz1c00568_si_001.pdf]

Supporting Information

Multimodal Microscale Imaging of Textured Perovskite-Silicon Tandem Solar Cells

*Elizabeth M. Tennyson,[1] Kyle Frohna,[1] William K. Drake,[1] Florent Sahli,[2] Terry Chien-Jen Yang,[2] Fan Fu,[2] Jérémie Werner,[2] Cullen Chosy,[3] Alan R. Bowman,[1] Tiarnan A. S. Doherty,[1] Quentin Jeangros,[2] Christophe Ballif,[2] Samuel D. Stranks[1,4]**

[1]Cavendish Laboratory, University of Cambridge, 19 JJ Thomson Avenue, Cambridge CB3 0HE, UK

[2]École Polytechnique Fédérale de Lausanne, Photovoltaics and Thin-Film Electronics Laboratory, Neuchatel, CH

[3]Department of Chemical Engineering, Stanford University, Stanford, CA 94305, US

[4]Department of Chemical Engineering & Biotechnology, University of Cambridge, Philippa Fawcett Drive, Cambridge CB3 0AS, UK

*Email: sds65@cam.ac.uk

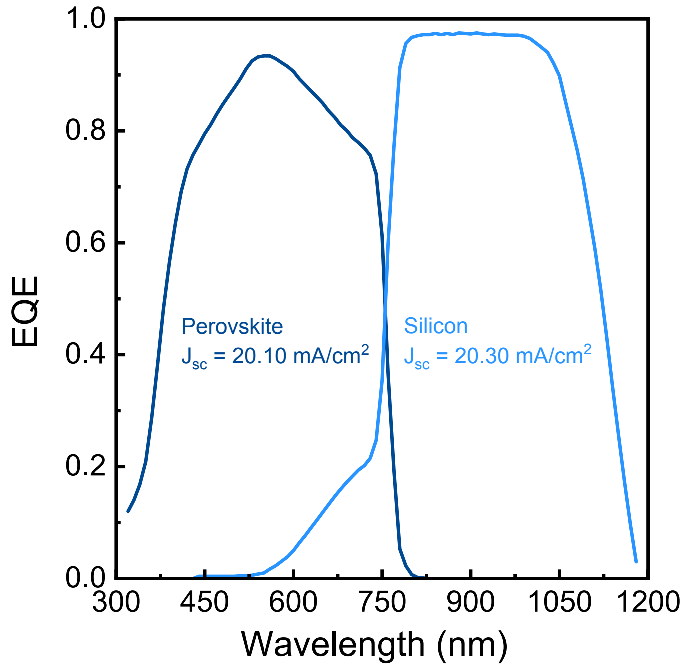

Figure S1. External Quantum Efficiency (EQE) measurement for the multi-junction solar cell.

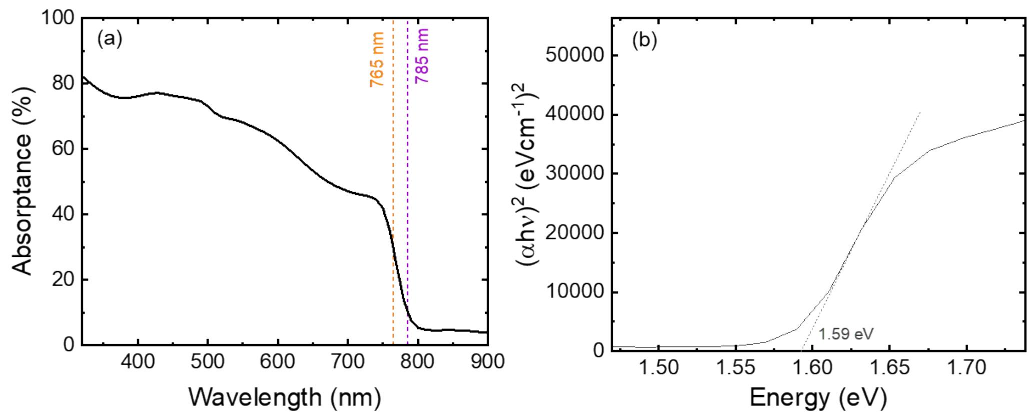

Figure S2. (a) UV-Vis spectrum displaying the macroscopic absorption profile of the perovskite film, with the two relevant emission wavelengths labelled. (b) Tauc plot of the perovskite sample, showing that the bandgap is 1.59 eV.

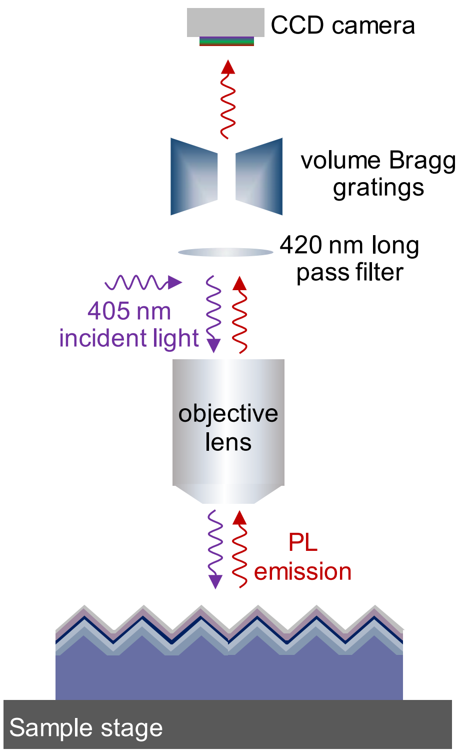

Figure S3. Schematic (not to scale) of the experimental setup for the wide-field hyperspectral PL microscope. A 405 nm laser excites the PL response in the perovskite, which is then spectrally and spatially resolved with the volume Bragg gratings and CCD camera, respectively. The sample stage is stationary throughout the data acquisition. During our measurements we use a 100× objective lens (NA = 0.9), with a spot size of 150 µm, and an area of uniform illumination of approximately 75 × 95 µm. The spatial resolution is diffraction limited.

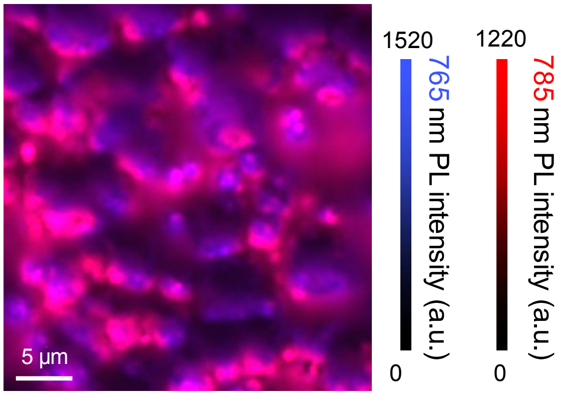

Figure S4. RGB image overlay of the PL emission intensity of λ_{em} = 765 nm (blue) and λ_{em} = 785 nm (red). The regions of magenta/purple indicate an overlap in PL emission.

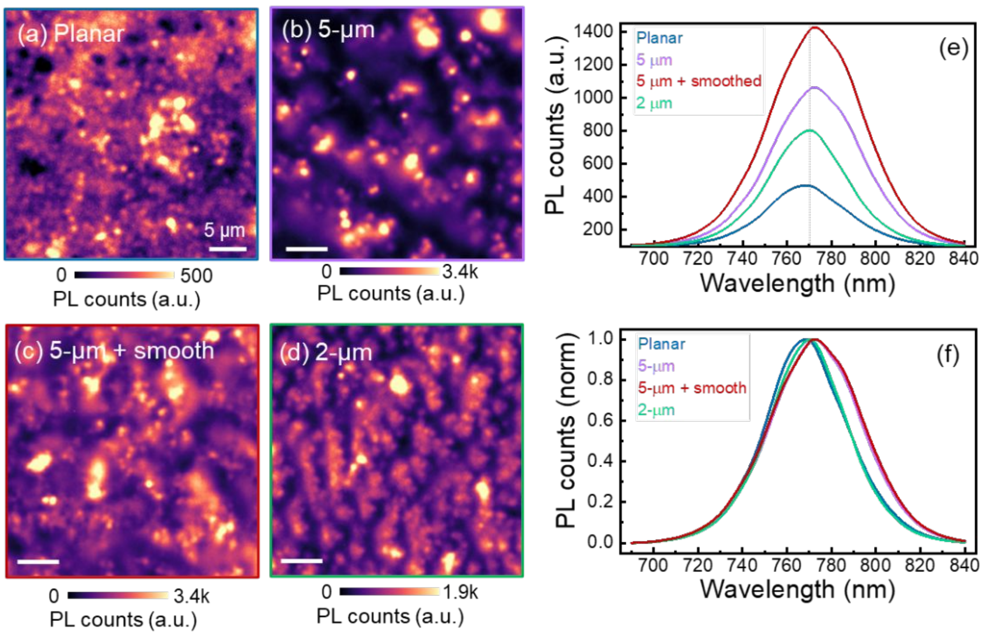

Figure S5. (a) Perovskite PL signal of a perovskite solar cell on planar c-Si device (with layer stack from bottom to top: Ag/ITO/ip/c-Si-flat/in/uc-Si(n)/uc-Si(p)/spiroTTB/PK/C_{60}/IZO) compared to (b-d) the perovskite PL response from a variety textured perovskite/c-Si tandem solar cells, all at λ_{em} = 770 nm. While on the planar sample the PL distribution is dominated by grain-to-grain variations, the emission of the texture samples are dominated by the Si pyramids. Spatially averaged PL spectra of the entire scan area for each sample (e) as absolute counts and (f) normalized. The perovskite composition was equivalent in all samples. The PL maps were taken in ambient conditions with a 100× objective, with 405-nm incident light (λ_{exc}), and a photon flux of 300 mW/cm^2. The identical excitation and collection conditions allows us to provide direct comparisons between the samples.

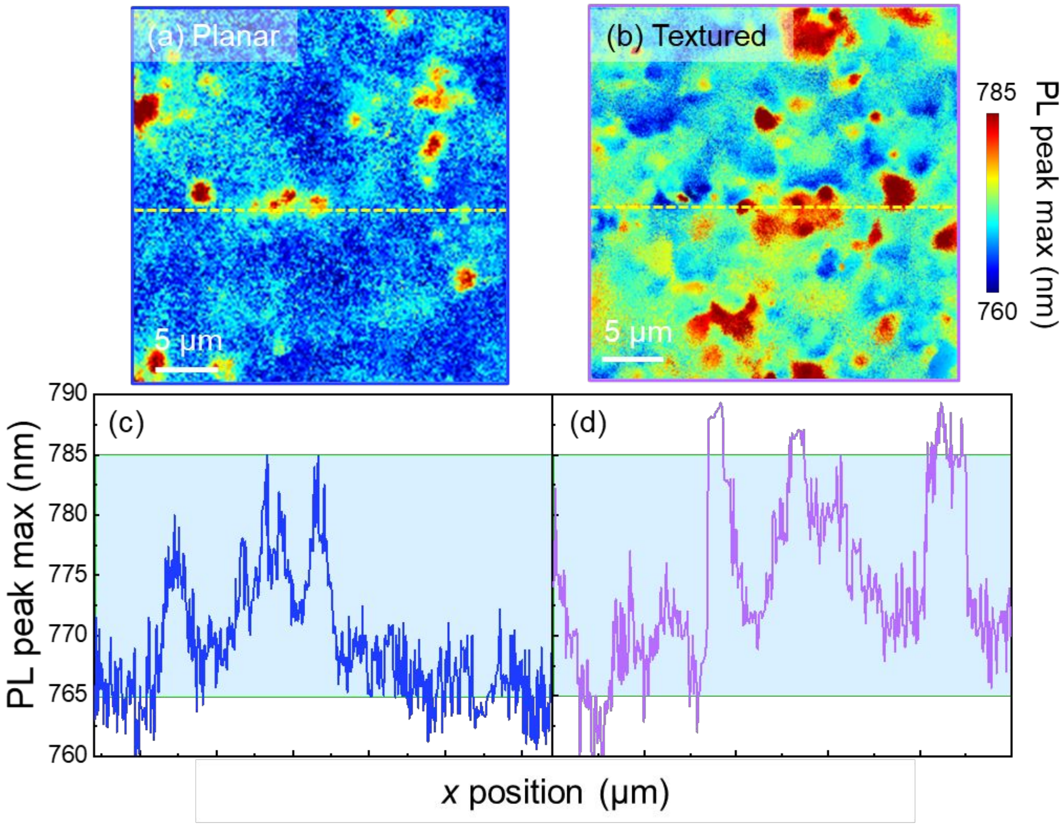

Figure S6. Perovskite PL emission maps on a (a) planar and (b) textured substrate at the same photon flux (60 mW/cm^2. (c,d) Line traces of the perovskite's PL emission as a function of *x* direction (from left to right) of the planar and textured sample. Here we highlight the differences in length scales and variability of the local PL emission fluctuations. The planar sample exhibits a grain-to-grain and minimal variation, while in contrast, the textured sample has large 3-10 μm variations in PL intensity.

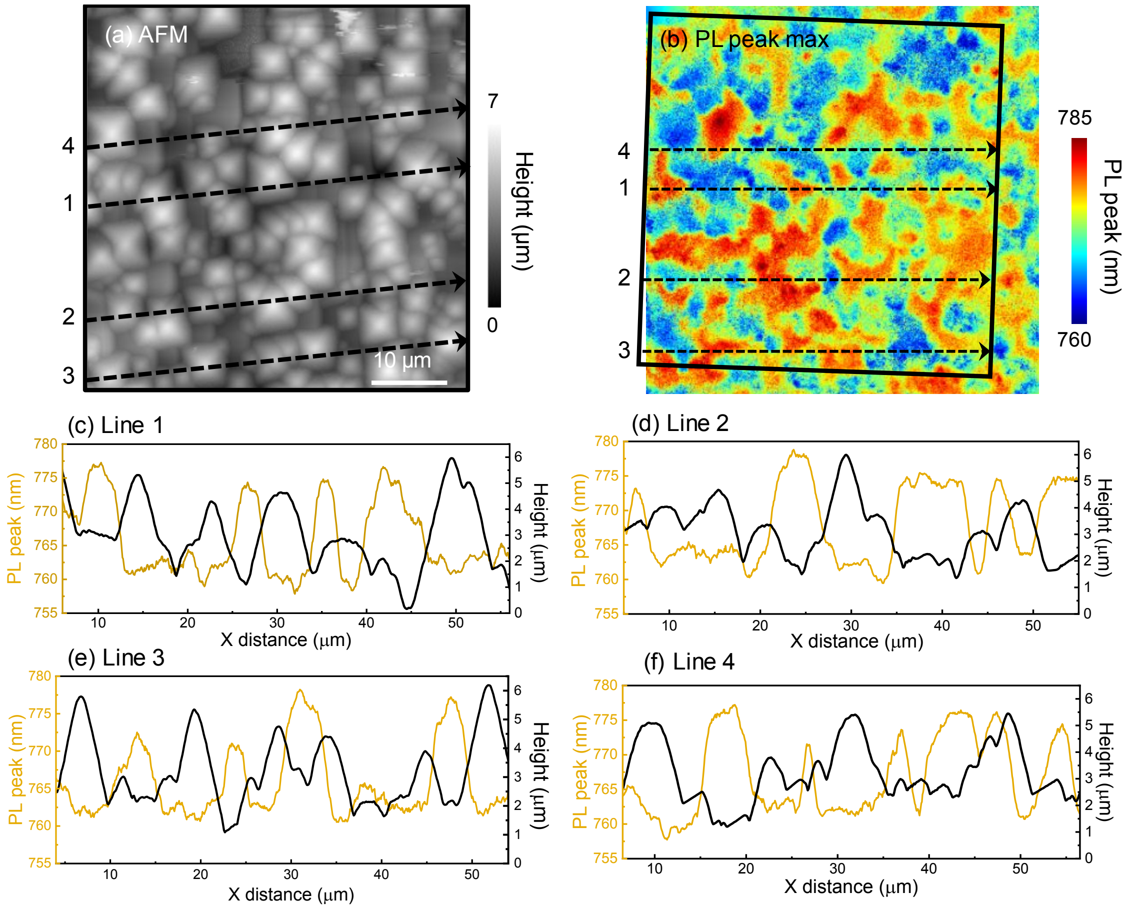

Figure S7. (a) 50 × 50 μm^2 AFM map of perovskite on textured Si, with four numbered line traces specified by black dashed lines. (b) The corresponding PL peak maximum map, with the black box indicating the AFM map region along with the same four line traces. (c-f) The four AFM line traces plotted with their correlated PL peak maximum position. Consistently, the valleys and sidewalls of the pyramids show longer wavelengths dominating the PL intensity, while the pyramid apexes emit with shorter wavelengths (i.e. higher energies).

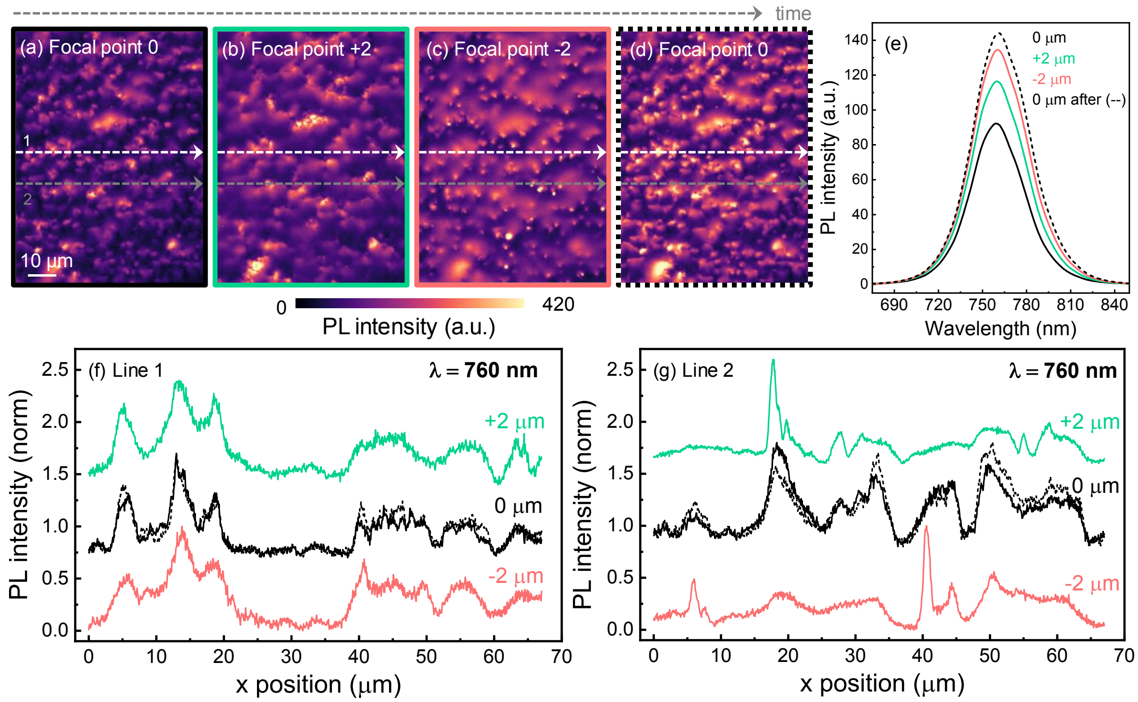

Figure S8. Wide-field PL maps at λ_{em} = 760 nm of perovskite on a textured multi-junction solar cell as a function of focal point: (a) focal point = 0 µm (b) +2 µm (c) -2 µm, and (d) 0 µm again. While there are light soaking effects during acquisition (time = 40 min for all four PL maps) as seen by plotting (e) PL intensity of the entire imaged region, the PL peak maximum position is maintained. The PL distribution, although visually it appears variable, when plotting the normalized PL intensity as a function of *x* position for multiple line scans in (f) and (g), we discover that the focal point at 0 µm is, in fact, a super-position of the two focal point extremes.

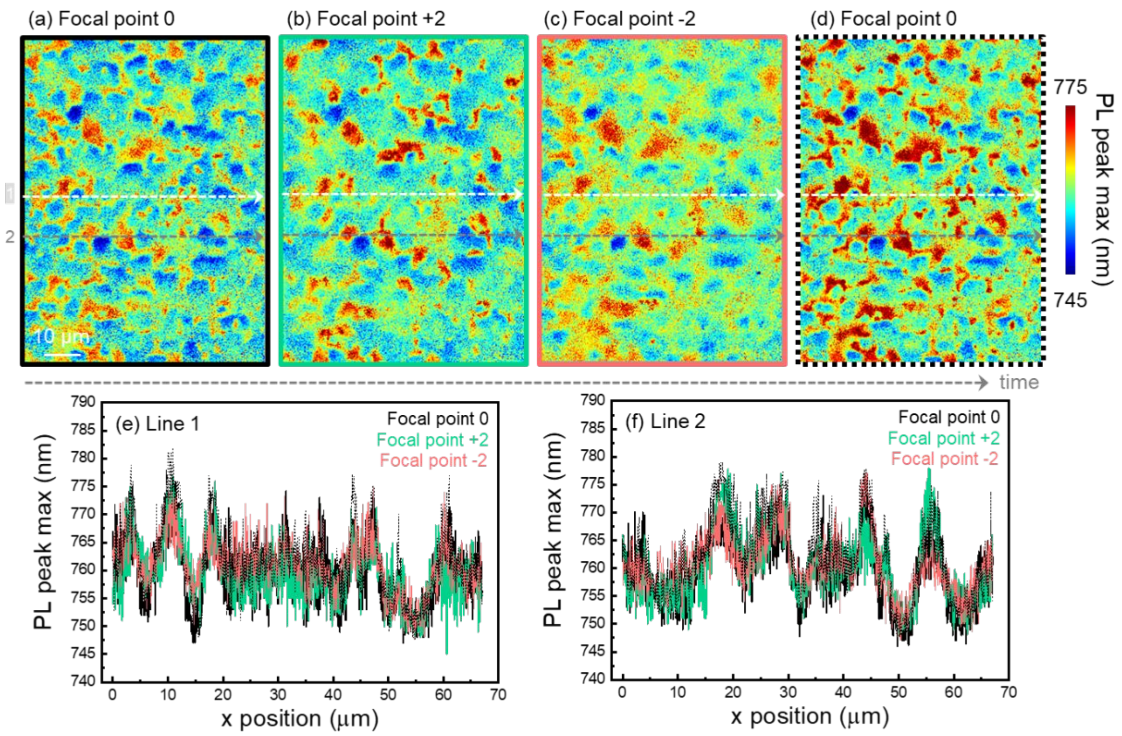

Figure S9. Wide-field PL peak maximum maps of perovskite on a textured tandem solar cell as a function of focal point: (a) focal point = 0 μm (b) +2 μm (c) -2 μm, and (d) 0 μm again. (e) and (f) are two representative line traces (same as in Figure S6) plotting the PL peak maximum as a function of position. From this we verify that the local peak position does not change as a function of focal point.

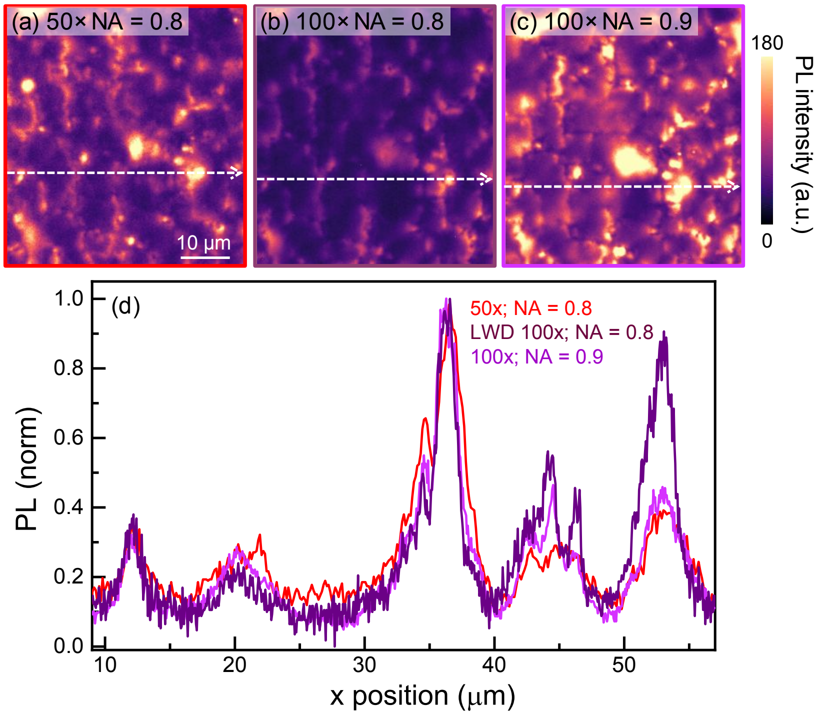

Figure S10. (a-c) Wide-field PL maps at λ_{em} = 770 nm of perovskite on a textured tandem solar cell as a function of focal point imaged with different objective lens magnification and numerical apertures (NA). (d) Normalised PL intensity of the line trace indicated in maps (a-c) as a function of position. The PL distribution is consistent despite differing objective lenses.

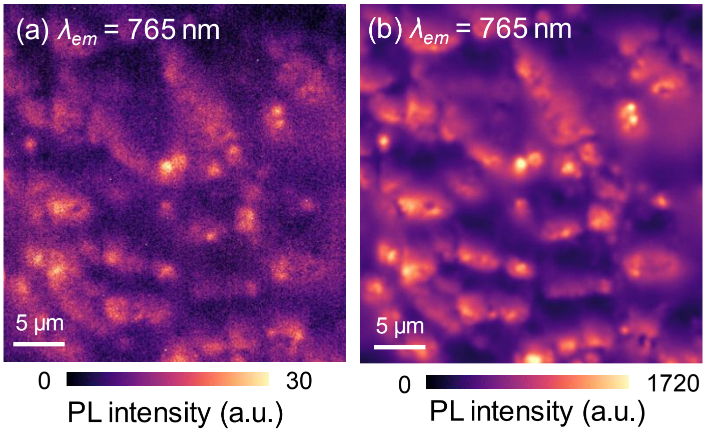

Figure S11. Perovskite PL emission under (a) 100 mW/cm^2 and (b) 2150 mW/cm^2 at λ_{em} = 765 nm. Here we see that the PL spatial distribution remains unchanged at higher incident power densities.

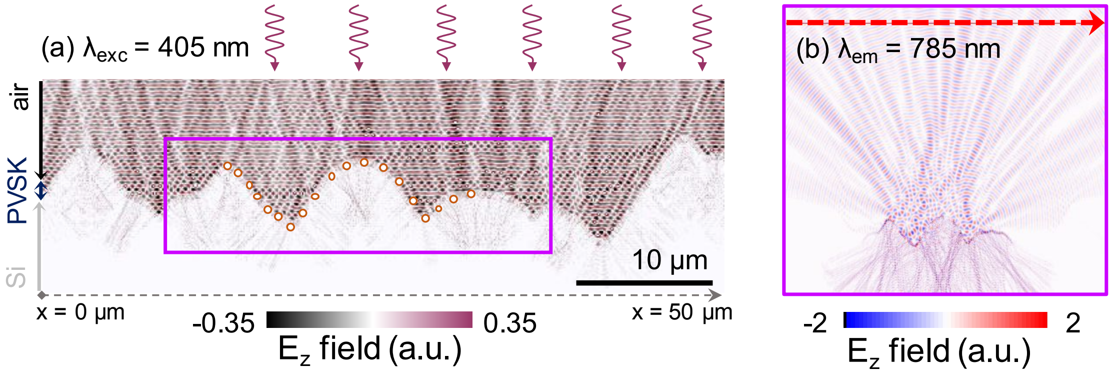

Figure S12. (a) Finite difference time domain (FDTD) calculation of λ_{exc} = 405 nm using the AFM line trace shown in Figure 2f of the main manuscript as an input for the model, colour scale represents the E_z field strength. The orange circles indicate where point sources were placed in (b) the FDTD simulation of perovskite photon emission. This calculation is only showing λ_{em} = 785 nm, the red dashed line indicates the monitor's position in the far field.

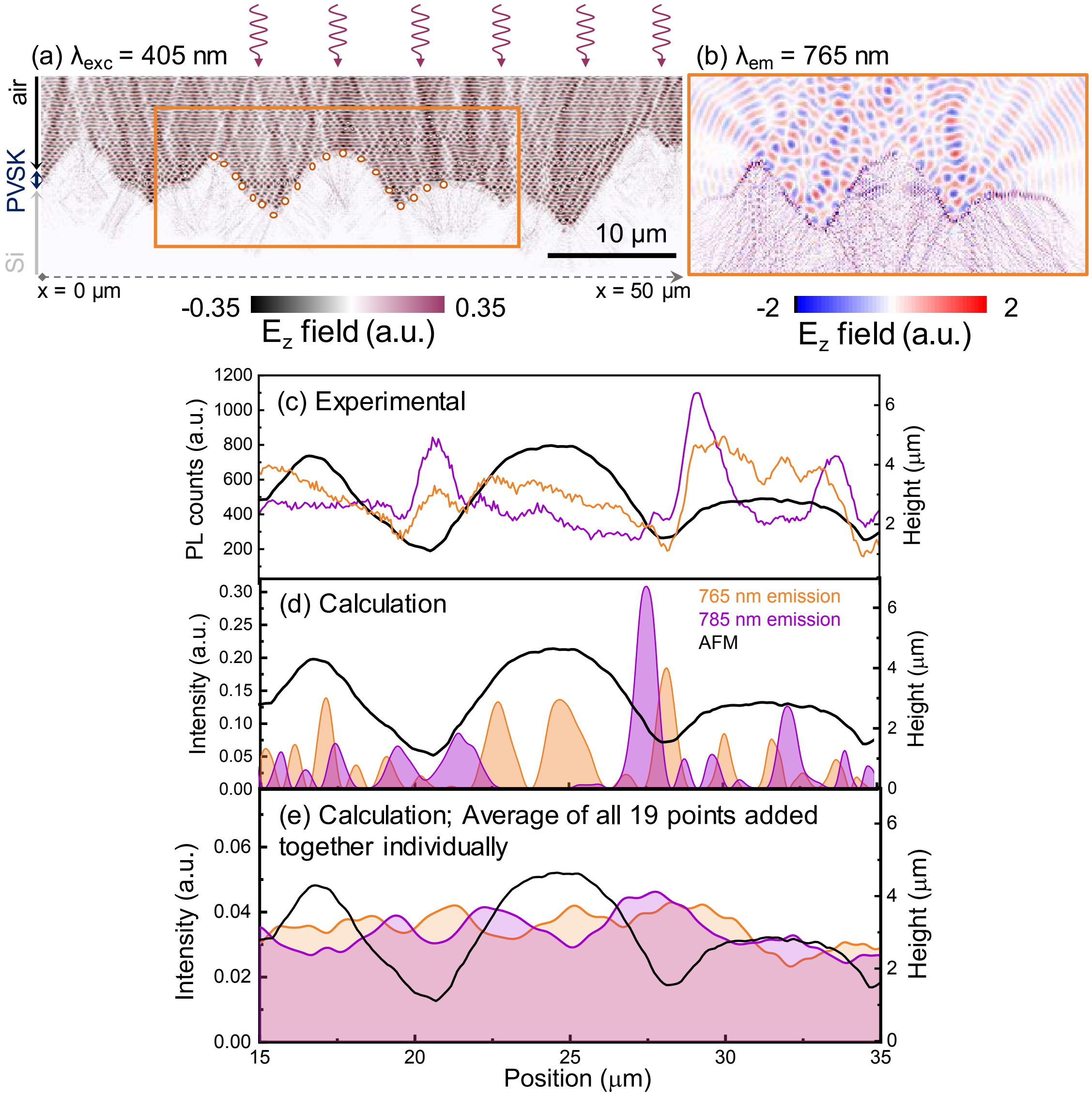

Figure S13. (a) Finite difference time domain (FDTD) calculation of λ_{exc} = 405 nm using the AFM line trace shown in Figure 2f of the main manuscript as an input for the model, colour scale represents the E_z field strength. The orange circles indicate where point sources were placed in (b) the FDTD simulation of perovskite photon emission. This calculation is only showing λ_{em} = 765 nm. (c) Optical microscopy PL results and (d),(e) FDTD emission calculations for 19 emission points distributed near the perovskite surface of perovskite emission λ_{em} = 765 (orange) and 785 nm (purple) where the intensity of the point emitters were detected either (d) all simultaneously or (e) added together individually. Good qualitatively agreement is found with both modes of calculation. The black lines representing the morphology from AFM in (c)-(e). A smoothing filter = 50 pts was added to both curves in (e) to reduce the number of oscillations, making the data easier to interpret by eye.

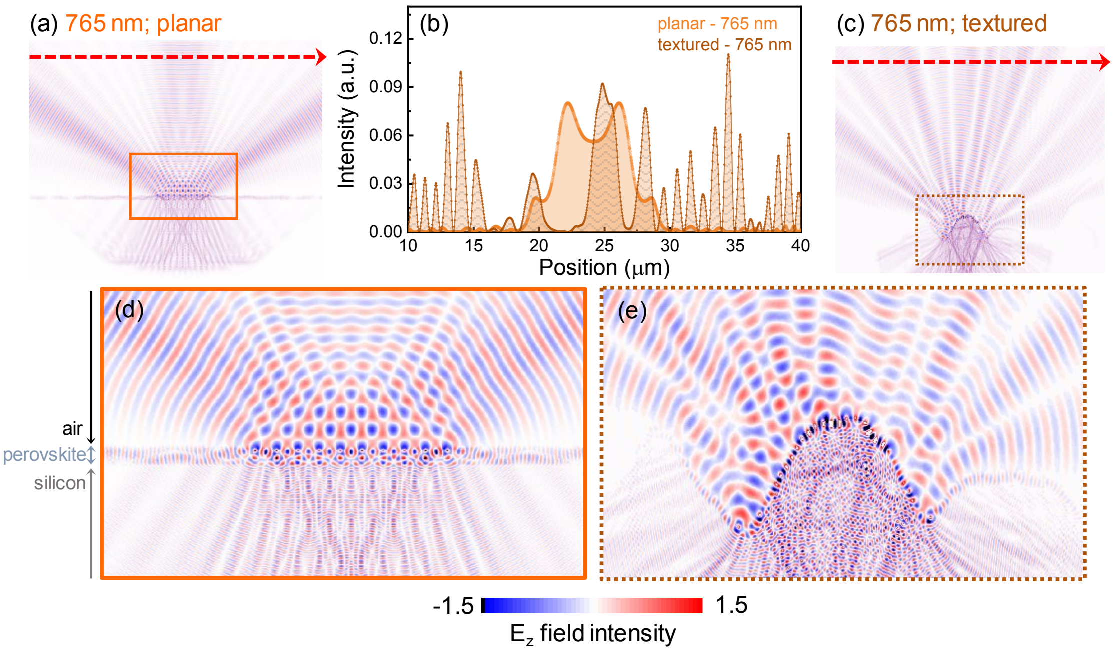

Figure S14. (a) Calculated E_z field intensity for a planar perovskite with thickness = 430 nm on top of Si, with 10 point emitters equally separated along the top surface of the perovskite layer. (b) The far-field photon intensity profile extracted from the dashed red line local from (a) and (c), showing the intensity for λ_{em} = 765 nm for the planar sample (orange) and the textured layer stack (dark orange, dotted). (d) Zoom in image of the planar and (e) textured λ_{em} = 765 nm E_z field outputs.

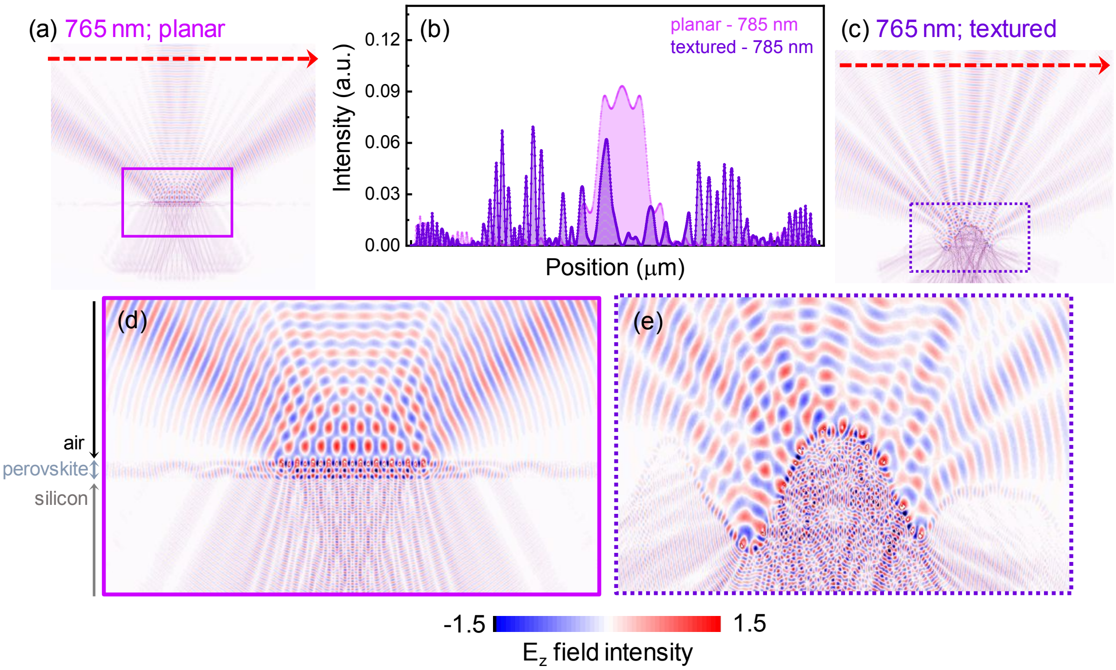

Figure S15. (a) Calculated E_z field intensity for a planar perovskite with thickness = 430 nm on top of Si, with 10 point emitters equally separated along the top surface of the perovskite layer. (b) The far-field photon intensity profile extracted from the dashed red line local from (a) and (c), showing the intensity for λ_{em} = 765 nm for the planar sample (purple) and the textured layer stack (dark purple, dotted). (d) Zoom in image of the planar and (e) textured λ_{em} = 785 nm E_z field outputs.

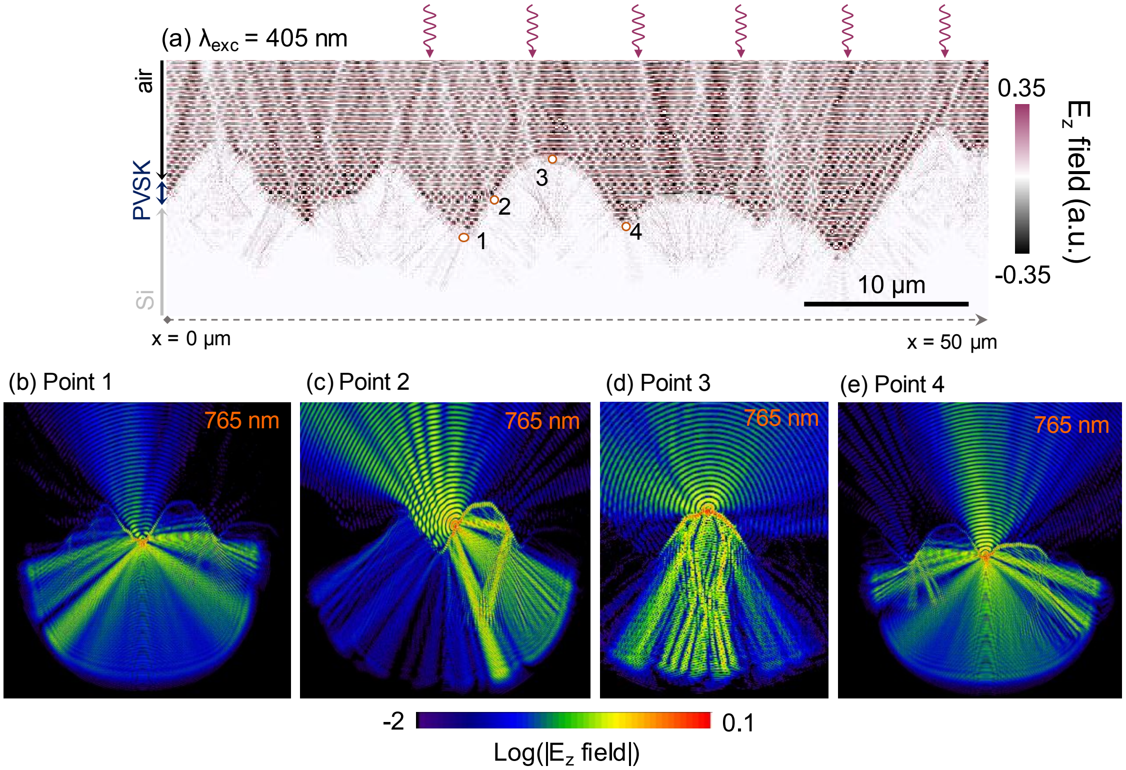

Figure S16. (a) Calculated E_z field intensity of incoming light λ_{exc} = 405 nm, for a textured perovskite with thickness = 430 nm on top of Si, displaying four point emitters placed along the top surface of the perovskite layer. (b-e) Point emitter number 1, 2, 3 and 4 showing a calculated log E_z field strength for λ_{em} = 765 nm.

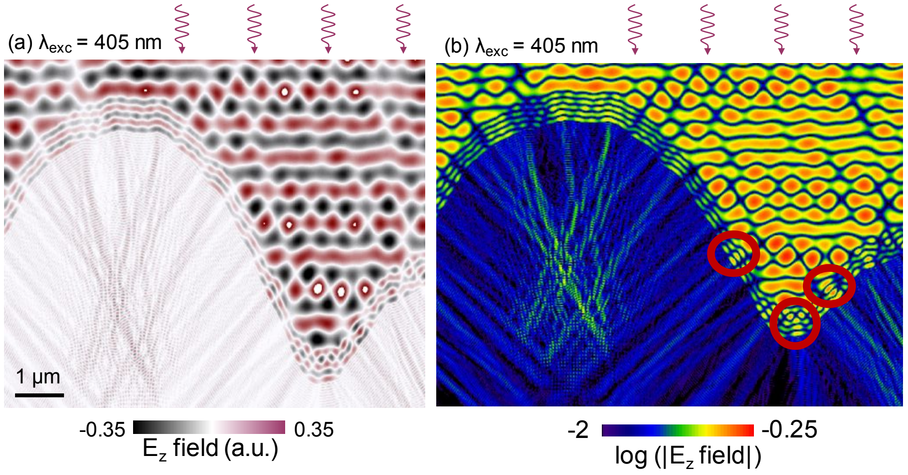

Figure S17. (a) Zoom-in of the FDTD calculation in Figure 3a of the main manuscript, with λ_{exc} = 405 nm, colour scale represents the E_z field strength. (b) The same region as in (a) but here the colour scale is the log of the absolute value of the E_z field to facilitate viewing the strength of the local absorption. We observe hot-spots of absorption in the perovskite layer (designated with red circles), which reside in valleys and lower sidewalls of the pyramid structure, indicating more intense photon interaction in these regions.

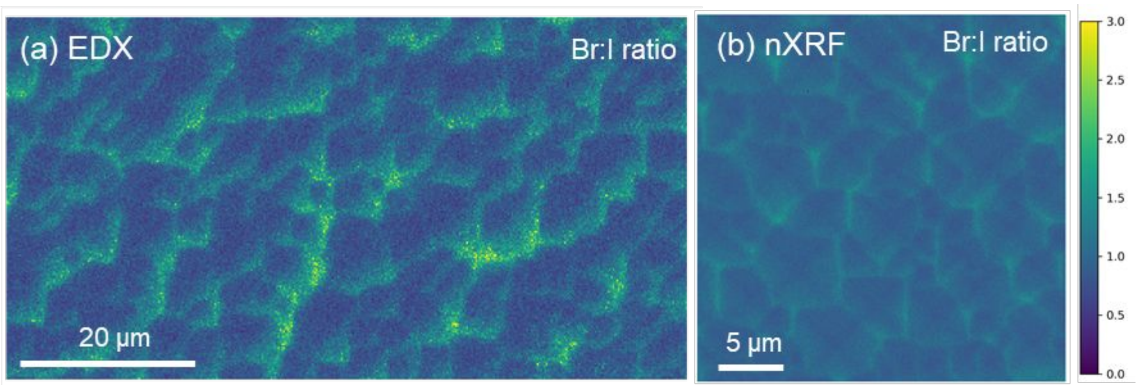

Figure S18. Chemical imaging of the Br:I halide ratio in a textured perovskite/c-Si tandem sample via (a) energy-dispersion x-ray (EDX) spectroscopy and (b) nanobeam X-ray fluorescence (n-XRF). Both maps show a slightly higher Br content in the pyramid valleys, for which we would expect a slight blue shift in the emission spectrum at the valley. However, in the hyperspectral wide-field PL maps we observe the opposite effect, (i.e. longer wavelength emission in the valleys) giving strong evidence that the texturing dominates the optoelectronic response over any fluctuations in the chemical composition. Both chemical maps are threshold between 0 and 3 after normalizing to their mean value.

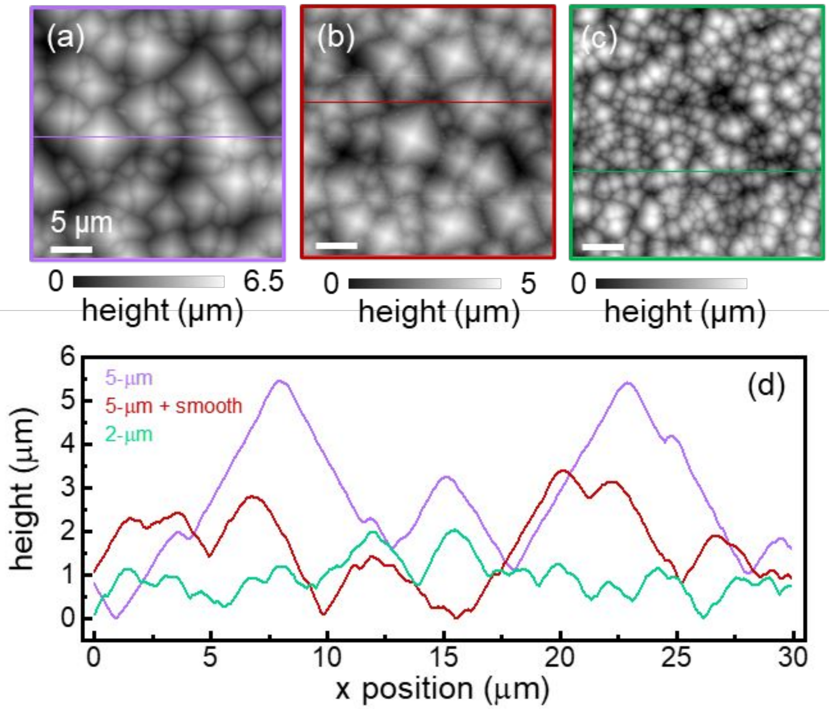

Figure S19. (a-c) 30 × 30 µm2 AFM maps of the three different perovskite/c-Si texturing schemes displaying the morphology of the 5 µm, 5 µm + smooth, and 2 µm geometries, respectively. (d) AFM line traces of the three texturing geometries displaying the distinct morphologies.

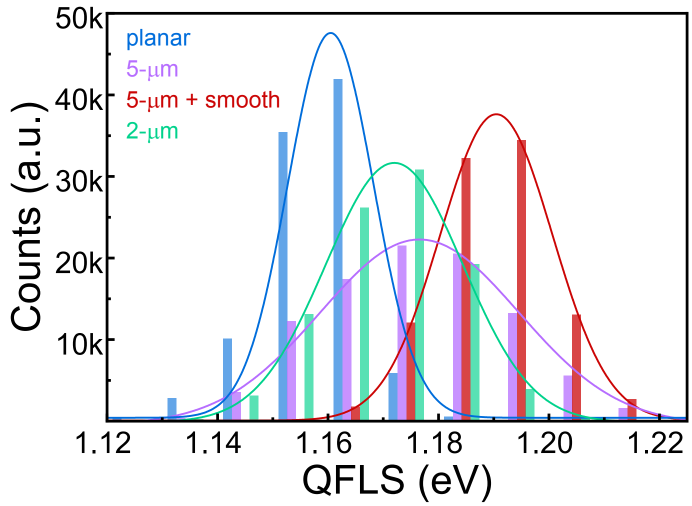

Figure S20. QFLS histograms for each etching schemes and the planar sample, shown here for reference, which shows a lower QFLS value with a narrower full-width half maximum. Each histogram was individually fit with a Gaussian peak with an R2 value of >0.99 in all cases.

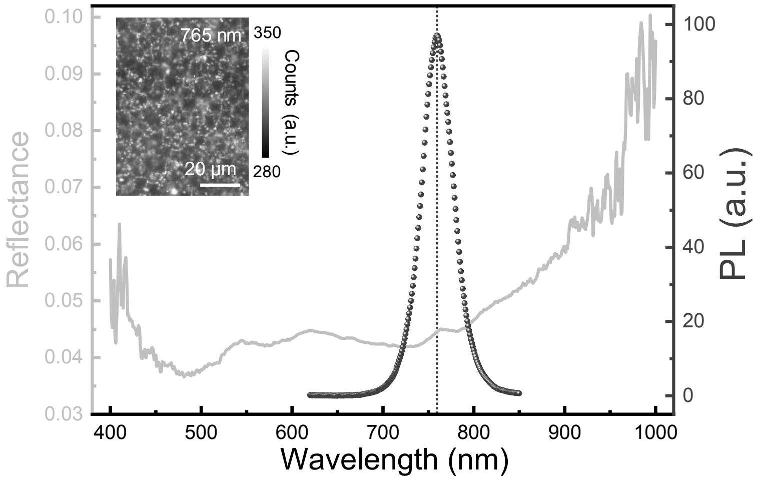

Figure S21. Perovskite average reflectance (left axis) and PL response (right axis) for an entire wide-field area of a textured perovskite/Si tandem solar cell. Here we see that the reflectance remains below 10% for the entire wavelength range used here. The reflectance data was acquired by measuring the reflection as a function of wavelength with the wide field microscope (see inset for reflection map at 765 nm), a dark reflection measurement (for background noise) and a calibrated mirror reflection measurement as a reference, then subsequently, dividing the two reflection measurements from one another. There is a grating change at 600 nm, and a grating correction is performed by the microscope software by acquiring reflection measurements with both gratings in the wavelength range = 580 – 620 nm.

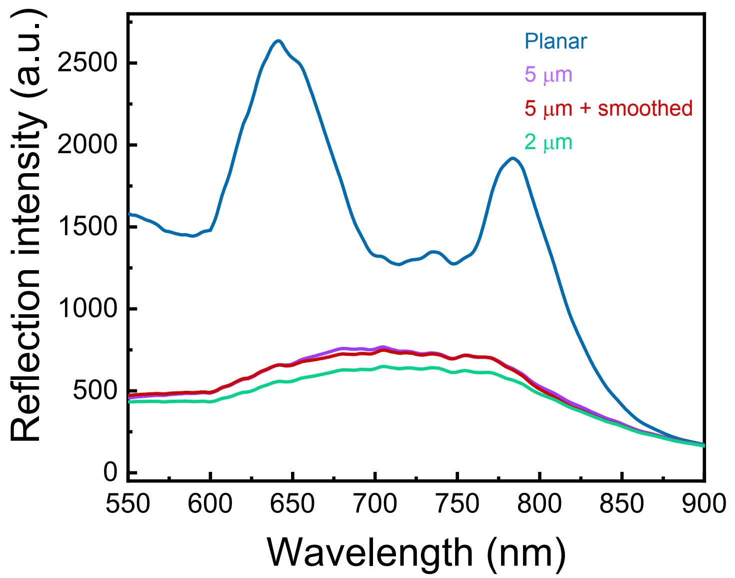

Figure S22. Average reflectance spectra of the multiple samples with different texturing, compared to the perovskite sample fabricated a planar c-Si device. The reflection spectra of textured surfaces are consistently lower than the planar sample, however, do not vary much between each other. All samples were measured with the white light on the hyperspectral microscope at the same lamp intensity.

Table S1. Optical parameters used in the FDTD MEEP simulations. Silicon values come from ref [1] and perovskite values are extracted from x = 0.2 in ref [2].

Optical property	405 nm	765 nm	785 nm
n_{Si}	5.47	3.70	3.69
k_{Si}	0.26	0.00697	0.00611
α_{Si}	8.067×10^4 cm^{-1}	1.145×10^3 cm^{-1}	9.78×10^2 cm^{-1}
n_{PVSK}	2.44	2.34	2.31
k_{PVSK}	0.65	0.027	0
α_{PVSK}	2.017×10^5 cm^{-1}	4.435×10^3 cm^{-1}	0

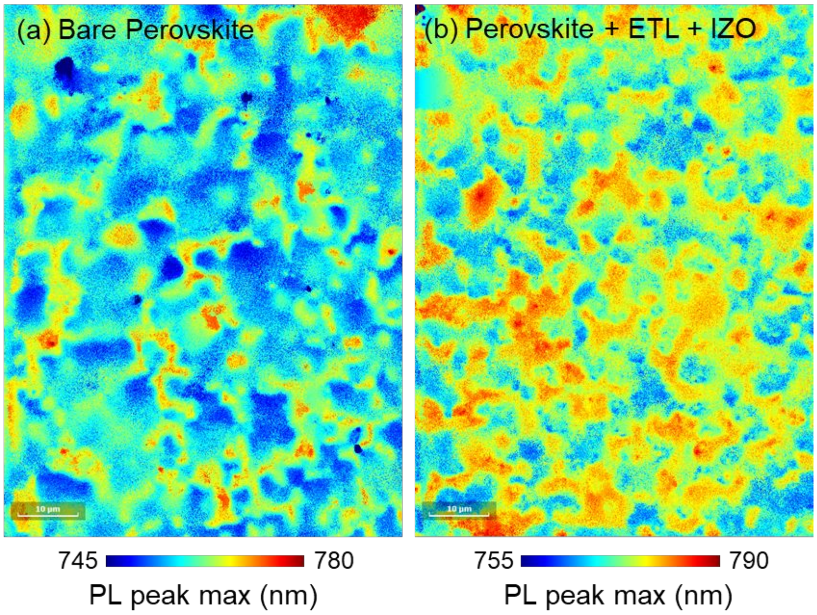

Figure S23. PL peak maximum maps of (a) a bare perovskite film conformally coated on textured c-Si and (b) perovskite, electron transport layers and IZO conformally coated on textured c-Si device. In both PL peak maximum, the same pattern which can be attributed to the underlying texture.

Supporting Information

References

(1) Green, M. A. Self-Consistent Optical Parameters of Intrinsic Silicon at 300K Including Temperature Coefficients. *Solar Energy Materials and Solar Cells* **2008**, *92* (11), 1305–1310.

(2) Tejada, A.; Braunger, S.; Korte, L.; Albrecht, S.; Rech, B.; Guerra, J. A. Optical Characterization and Bandgap Engineering of Flat and Wrinkle-Textured FA0.83Cs0.17Pb(I1–XBrx)3 Perovskite Thin Films. *Journal of Applied Physics* **2018**, *123* (17), 175302.
